# Sport-Based Exercise in Pediatric Acquired Brain Injury: Protocol for a Randomized Controlled Trial

**DOI:** 10.3390/jcm14175970

**Published:** 2025-08-23

**Authors:** Andrea Gutiérrez-Suárez, Marta Pérez-Rodríguez, Agurtzane Castrillo, Javier Pérez-Tejero

**Affiliations:** 1 Department of Physiotherapy, Medicine and Biomedical Sciences, Faculty of Physiotherapy, Universidad de A Coruña, 15071 A Coruña, Spain; 2 Segunda Parte Foundation, 28034 Madrid, Spain; 3 Department of Health and Human Performance, Faculty of Physical Activity and Sport Science-INEF, Universidad Politécnica de Madrid, 28040 Madrid, Spain

**Keywords:** acquired brain injury, adolescents, participation, quality of life, sports

## Abstract

**Background/Objectives**: Pediatric acquired brain injury (ABI) often results in persistent challenges that extend beyond motor impairments, affecting quality of life (QoL), social participation, and engagement in physical activity. Given the complexity and chronicity of these outcomes, there is a pressing need for multidimensional interventions grounded in the International Classification of Functioning, Disability and Health (ICF). Sport-based exercise interventions, when developmentally adapted and tailored to individual interests, may promote intrinsic motivation, peer connection, and sustainable engagement—factors especially relevant in pediatric ABI populations, who often experience reduced physical activity and social isolation. However, standardized, replicable protocols specifically tailored to this population remain scarce. This study presents the protocol for a randomized controlled trial evaluating the effects of a 16-week sport-based intervention on QoL, social participation, physical activity engagement, and motor functioning tailored for adolescents with pediatric ABI. **Methods**: Participants will be randomly assigned to an intervention group or a control group receiving usual care. The intervention consists of one weekly 60-minute session, led by trained professionals in adapted physical activity and pediatric neurorehabilitation. It combines sport-based motor skill training, cooperative games, and group activities specifically tailored to each child’s developmental level, motor abilities, and preferences. Outcomes will be assessed at baseline and following the 16-week intervention period, focusing on QoL, participation, physical activity engagement, and motor functioning. **Discussion:** This study introduces a structured, child-centered model that bridges clinical rehabilitation and community-based sport. By integrating motor and psychosocial targets through a group sport-based intervention, it aims to enhance recovery across ICF domains. Findings may inform interdisciplinary practice and support the development of sustainable strategies to promote long-term engagement and well-being in adolescents with ABI.

## 1. Introduction

Pediatric acquired brain injury (ABI) refers to any non-congenital brain damage after birth in adolescents with typical neurodevelopment, resulting in a disruption of neural function [1,2]. It encompasses diverse etiologies, including traumatic brain injury, stroke, infections, and tumors [1,2].

ABI is a leading cause of disability among the pediatric population, producing complex, long-lasting impairments across all domains of the International Classification of Functioning, Disability and Health (ICF) [3,4,5]. These impairments negatively affect quality of life (QoL), limit participation, and impact physical well-being [6,7]. Consequently, adolescents with ABI require sustained support from families, educational systems, and healthcare services, imposing significant societal and economic burdens [8,9].

The multidimensional and chronic nature of pediatric ABI requires a comprehensive, interdisciplinary, and holistic approach that extends beyond conventional therapies focused primarily on physical recovery, to encompass psychosocial dimensions critical for global well-being [10,11,12,13].

In this context, exercise interventions grounded in sports have recently gained attention for their ability to simultaneously target multiple health domains in neurological populations [12,14,15,16,17]. It combines complex motor skills, cognitive engagement, and social interaction, providing a multifaceted stimulus that promotes neuroplasticity and functional improvements across different areas [12,14,15,16,17]. Particularly in pediatric populations, growing evidence highlights sport-based exercise as a promising approach to enhance motor recovery while also targeting emotional, cognitive, and behavioral sequelae [18,19]. Evidence suggests that sport-based exercise induces neurophysiological changes beneficial to executive function, mood regulation, and attention—all commonly affected in pediatric ABI [20].

Furthermore, participation in group-based exercise sessions tailored to each participant’s developmental stage and interests promotes peer interaction, self-esteem, and social engagement—factors especially relevant for adolescents who frequently experience isolation and reduced physical activity participation [21,22,23,24]. This approach also enhances intrinsic motivation, facilitating long-term adherence to active, socially integrated lifestyles, reinforcing gains across multiple ICF domains [22,23,24]. However, despite these potential benefits, there remains a lack of standardized and replicable sport-based protocols specifically designed for adolescents with ABI that explicitly integrate the ICF framework—particularly those linked to psychosocial well-being.

Therefore, the aim of the present study is to design and evaluate a sport-based exercise intervention tailored to the specific needs of adolescents with ABI. The program is tailored to the developmental characteristics and functional needs of this population, with the goal of promoting improvements in quality of life, social participation, motor function, and physical activity engagement.

## 2. Materials and Methods

This study was developed in accordance with the Standard Protocol Items: Recommendations for Interventional Trials (SPIRIT) guidelines [25]. It has been registered on Clinicaltrials.gov (Identifier: NCT06804486) and received ethical approval from the Regional Ethics Committee of Madrid (IRB approval no. EC 33.25).

### 2.1. Trial Design and Setting

The study protocol describes the methods for a controlled trial with two parallel groups. The trial will compare the effectiveness of a sport-based exercise program in combination with usual care (experimental group), versus usual care alone (control group) in adolescents with ABI. The study screening, intervention, and assessments will be carried out at a pediatric hospital in Madrid, Spain, called Niño Jesús Hospital.

**Figure 1:**
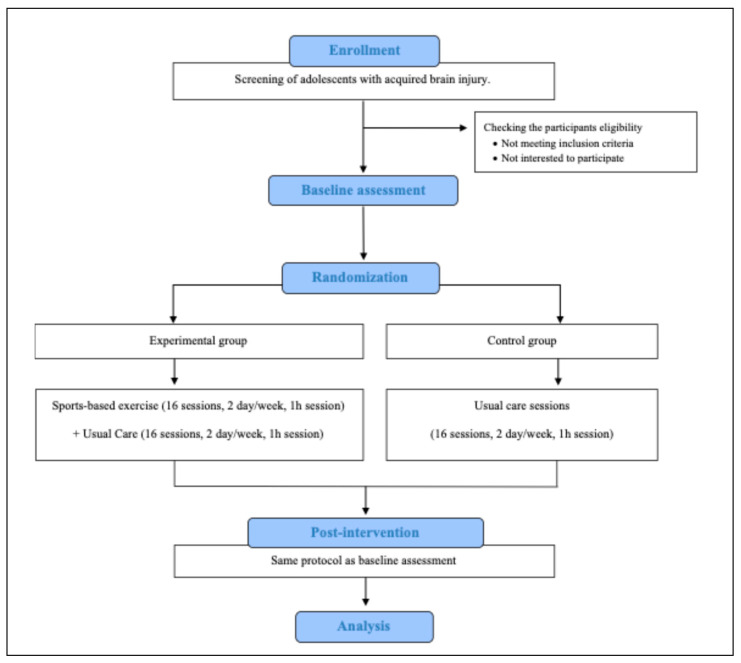
Flowchart.

A flowchart of the study design according to the CONSORT guidelines is shown in Figure 1.

### 2.2. Eligibility and Recruitment

The study will involve adolescents aged 11 to 17 years with a confirmed diagnosis of ABI, regardless of etiology, who are receiving care at the Pediatric ABI Unit of Hospital Niño Jesús. Participants may be either inpatients or outpatients and may include individuals using wheelchairs or other mobility aids. A non-probabilistic, convenient sampling approach will be used based on participant availability and interest. Healthcare professionals within the Pediatric ABI Unit will identify eligible participants according to the inclusion and exclusion criteria outlined in Table 1. Recruitment will be conducted over a three-month period. All eligible participants and their legal guardians will provide written informed consent after receiving detailed information about the study’s objectives and procedures.

### 2.3. Allocation and Blinding

Participants will be randomly allocated to either the intervention or control group using centralized, independent, stratified block randomization. A computer-generated random number sequence will ensure a 1:1 allocation ratio. Blinding will be maintained for assessors.

### 2.4. Interventions

#### Sport-Based Exercise and Usual Care (Experimental Group)

Participants in the experimental group will engage in a sport-based exercise program in addition to their usual hospital care sessions. The program consists of twice-weekly sessions, each lasting 60 min, over a period of three months. It is structured around a sport-based framework that integrates motor patterns and task demands from a variety of sports disciplines. Sessions are organized in a circuit format that emphasizes functional movement, progressive task complexity, and social interaction within small groups of two to four participants, grouped by age and functional capacity. Each session follows a sample structure including warm-up activities (e.g., dynamic stretching and basic motor skill drills), main circuit stations featuring sports-based tasks such as adapted ball games, obstacle navigation, and coordination drills, and a cool-down with relaxation exercises.

Session content is continuously tailored to each participant’s motor abilities to enable individualized progression, including specific adaptations such as seated or supported activities for children with limited mobility, simplified task demands for those with cognitive or behavioral challenges, and use of assistive devices as needed. Participants’ individual interests and functional goals are identified through baseline assessments and ongoing feedback, which guide activity selection and progression. All sessions are supervised to ensure accuracy and safety.

The Template for Intervention Description and Replication (TIDieR) checklist was used to ensure complete reporting of the intervention (Table 2) [26].

#### Usual Care (Control Group)

Participants in the control group will continue to receive their usual care for the duration of the study and will be reassessed. Usual care includes a range of therapies (e.g., physiotherapy or occupational therapy) provided by the hospital services. After the post-intervention assessment (3 months after the intervention), participants in this group will have the opportunity to attend the same program sessions if they wish.

### 2.5. Harms

Potential risks will be assessed prior to the start of the study and monitored continuously throughout the trial. Any adverse events, particularly serious or unexpected ones, will be documented by the research team in accordance with standard protocols and reported to the ethics committee. Safety monitoring will apply to both study groups, ensuring that any unintended effects are identified and addressed appropriately.

### 2.6. Outcome Measures

#### 2.6.1. Sociodemographic, Clinical, and Anthropometric Data

A structured baseline questionnaire will be administered to collect sociodemographic (age, sex, school type), clinical (primary diagnosis, years since diagnosis, laterality, current therapies, comorbid conditions), and anthropometric data (height and weight to calculate BMI in kg/m^2^). Additional screening items will assess levels of consciousness and orientation, potential contraindications for physical activity, prescribed medications, and relevant comorbidities (e.g., epilepsy, asthma).

#### 2.6.2. Pediatric Quality of Life Inventory (PedsQL)

A 23-item questionnaire that quantifies health-related quality of life across four domains (physical, emotional, social, and school). Scores for each domain and the total scale are the mean of item responses, with higher values indicating better quality of life. Participants self-report; parents provide proxy ratings for younger participants. The PedsQL shows good reliability and validity in cohorts with ABI, brain tumors, and traumatic brain injury [27].

#### 2.6.3. Child and Adolescent Scale of Participation (CASP)

A 20-item scale that rates participation in home, school, and community settings on a four-point Likert scale. Scores are expressed as a percentage of full participation; lower percentages indicate greater restriction. The CASP has been validated and widely used in intervention studies involving pediatric ABI [28].

#### 2.6.4. Global Physical Activity Questionnaire (GPAQ)

A 19-item instrument that captures moderate- and vigorous-intensity physical activity undertaken at work, during transport, and in leisure. Minutes per day in each intensity band are converted to metabolic-equivalent minutes to yield a total physical-activity score. The GPAQ demonstrates acceptable test–retest reliability and low-to-moderate criterion validity versus accelerometry in pediatric samples [29].

#### 2.6.5. Bruininks–Oseretsky Test of Motor Proficiency, Second Edition (BOT-2)

A test of gross motor proficiency through five subtests: bilateral coordination, balance, running speed and agility, upper-limb coordination, and strength. Raw scores are converted into scaled scores and composite scores for each motor domain and total score. The BOT-2 demonstrates high test–retest reliability and has been validated in pediatric populations with brain tumors, traumatic brain injury, and developmental coordination disorder [30].

### 2.7. Study Timeline

Participants will undergo baseline assessments prior to randomization, followed by a second evaluation at the end of the 3-month intervention period (completion). The full study timeline is detailed in Table 3.

### 2.8. Sample Size

The sample size estimation was based on the PedsQL, a validated outcome measure in pediatric neurological populations, including brain injury. Varni et al. (2006) reported standard deviations of approximately 10–11 points in cerebral palsy [31]. Assuming an SD of 10 points, with a two-sided alpha level of 0.05 and statistical power of 80%, the calculated sample size to detect a between-group difference is approximately 39 participants per group. Adjusting for an anticipated attrition rate of 15%, the final sample size was set at 45 participants per group.

### 2.9. Data Management and Monitoring

Data will be collected electronically using a secure, encrypted online database designed for each phase of the study, including eligibility screening and pre- and post-intervention assessments. No personally identifiable information will be stored in the database. Each participant will be assigned a unique identification code, generated through a random number sequence and stored separately in an independent table, ensuring a pseudonymized process. Data access will be limited to a secure hospital computer, with only the main investigators authorized to retrieve or manage the information. This system safeguards confidentiality and ensures protocol compliance.

### 2.10. Statistical Methods

The comparability of the study groups will be assessed by the similarity of the distribution of the variables of interest at baseline. The analysis will follow standard methods for randomized controlled trials, using two-group comparisons for all participants on an intention-to-treat basis. The chi-squared test or Fisher’s exact test will be used to compare proportions. Student’s t-test will be used to compare means between groups with normally distributed data. The Mann–Whitney test will be used to compare quantitative variables between groups in the case of a non-normal distribution, as determined by the Kolmogorov–Smirnov test. Correlations between quantitative measures will be determined by Spearman’s rho correlation coefficient. Matched pair data analysis is also calculated. In addition, multivariate analysis will be performed using multiple linear regression and logistic regression, depending on the response under consideration. This will allow for adjustment of the effectiveness of the intervention for potential confounders and the identification of other variables associated with each of the outcomes. Statistical significance will be set at ≤0.05. Data will be analyzed using the Statistical Package for the Social Sciences (SPSS) software, version 27.0 (IBM Corp, Armonk, New York, NY, USA).

## 3. Discussion

This protocol presents the rationale and design of a randomized controlled trial to evaluate the effects of a sport-based intervention tailored specifically for pediatric ABI, compared to usual care.

Unlike conventional pediatric rehabilitation focused predominantly on physical impairments, this intervention embraces a multidimensional framework addressing psychosocial well-being and participation—domains often overlooked despite their importance for sustained recovery [32,33].

By adapting sport-based activities to adolescents’ developmental levels and individual interests, the program addresses key determinants of adherence and social engagement in pediatric disability populations [21,22,23,24,33,34]. This child-centered and context-sensitive approach aligns with current best-practice recommendations advocating therapies that extend beyond impairment-focused models [33,34,35]. Moreover, delivering the intervention in a group setting further enhances intrinsic motivation, fosters peer interaction, and supports emotional regulation—well-documented effects in adolescents with neurological and developmental conditions [21,22,23,24].

Similar sport-based interventions have shown positive outcomes in pediatric populations with comparable profiles. In children with cerebral palsy, sport and exercise programs have been associated with improvements in cardiorespiratory fitness, gross motor function, and social participation [22]. Likewise, in developmental disabilities, structured sport-based interventions have demonstrated benefits in physical functioning and behavioral regulation [23,24,36]. These findings support the relevance of sport-based approaches in pediatric ABI, given overlapping functional and psychosocial challenges across these populations.

Different contextual and methodological factors warrant consideration within the scope of this protocol. While the intervention is designed to be flexible and developmentally appropriate, variability in participant age and functional status may affect engagement and the comparability of outcomes. Additionally, external influences such as transport access, family or caregiver involvement, and the level of support from educational or community settings may impact participation. These factors will be monitored and documented to inform the potential adaptation, scalability, and real-world applicability of the intervention.

As a study protocol, this trial introduces a replicable, theory-based intervention that addresses key gaps in pediatric neurorehabilitation literature. It may offer a transferable model to support the transition from clinical rehabilitation to community-based sport participation, encouraging active lifestyles and social inclusion from early recovery. Findings from this trial may have the potential to inform interdisciplinary clinical practice by providing a structured, child-centered approach aligned with family priorities and contextual demands. Ultimately, the results may help to guide clinical decision-making and facilitate the design of long-term engagement strategies that promote sustained participation and well-being in pediatric ABI.

## Figures and Tables

**Table 1:** Eligibility criteria.

Inclusion Criteria	Exclusion Criteria
Be aged <18 years at study entry.	Do not have a completed and signed informed consent form.
Have a medical diagnosis of acquired brain injury in the subacute or chronic stage.	Have medical comorbidities that contraindicate safe exercise (e.g., cardiac or respiratory instability, and uncontrolled seizures).
Be able to understand simple instructions from the program.	Failure to cooperate during the preliminary tests of the program.

**Table 2:** Template for intervention description and replication checklist for the intervention.

Items	Description of Items
Name of the intervention for the experimental/comparison group	Experimental group: sport-based exercise program and usual care sessions. Control group: usual care sessions.
Rationale	
Materials used in the intervention	Experimental group: The program is centered around different sports disciplines, which are adapted to participants’ functional condition, as well as different generic equipment, such as balls, chairs, tables, and platforms, among others.
Intervention procedures	Experimental group: The content of this program is designed around a sport-based framework incorporating motor patterns and task demands from various sports disciplines. It uses a circuit format to promote functional movement, progressive task complexity, and social interaction in small groups tailored by age and functional ability. Group composition will consider motor proficiency, cognitive, and behavioral profiles to balance skill levels and promote peer support. Strategies such as individualized task modification, use of visual and verbal cues, and structured routines will support engagement. Control group: Usual care encompasses a range of therapies (e.g., occupational therapy, physiotherapy, psychological support) provided by the rehabilitation services within the center. Typical frequency is 2–3 sessions per week, mainly individual or small group-based, and does not routinely include sport-based or structured group exercise. Following the post-intervention assessment, participants from this control group can participate in the same program sessions upon request.
Provider	Experimental group: The program will be delivered by a professional with expertise in neurological disorders and adapted physical exercise. Control group: Usual care for this group will be provided by professionals from the rehabilitation center.
Mode of intervention delivery	Experimental group: Presential, group-based sessions (from two to four participants). Control group: Presential, individual sessions.
Setting of intervention	Screening, interventions, and assessments will be conducted at the pediatric hospital, located in Madrid, Spain.
Dosage	Experimental group: Participants from this group will receive a 3-month exercise program which consists of sixteen sessions, 1 h session/day for 2 days/week. Control group: This group will continue with their usual care as usual 2 days/week, over 3 months, and then will be reassessed.
Tailoring	The session’s content will be continuously tailored and adapted by a research team to meet the individual needs and functional levels of each participant, thereby ensuring precise execution and security.
Modifications	Not applicable.
Fidelity assessment	The team responsible for delivering and managing the intervention will conduct a phone follow-up every 2 weeks, as well as other strategies to monitor the adherence and quality of the intervention (session attendance, fidelity checklists). Additionally, this professional will track the progress of participants from the control group during these follow-up calls.

**Table 3:** Schedule of enrolment, interventions, and assessments from the SPIRIT guidelines.

	Study Period		
	Allocation	Baseline	Completion
*ENROLLMENT:*			
Eligibility screen	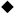		
Informed Consent	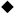		
*INTERVENTIONS:*			
Sports-based exercise + Usual Care		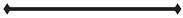	
Usual care		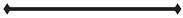	
*ASSESSMENTS:*			
Sociodemographic, anthropometric, and comorbidity data		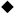	
Health-related quality of life (PedsQL)		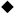	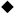
Physical Activity (GPAQ)		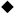	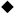
Participation (CASP)		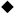	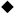
Motor functioning (BOT-2)		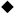	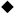

Abbreviations: PedsQL, Pediatric Quality of Life Inventory; CASP, Child and Adolescent Scale of Participation; GPAQ, Global Physical Activity Questionnaire; BOT-2, Bruininks–Oseretsky Test of Motor Proficiency.

## Data Availability

The datasets are available from the corresponding author on request.
